# The Psychological Impact of Rhino-Orbital Mucormycosis During the Second Wave of COVID-19 Pandemic From South East Asian Country

**DOI:** 10.7759/cureus.35349

**Published:** 2023-02-23

**Authors:** Shruti Srivastava, Nitika Beri, Gopal K Das, Pramod K Sahu, Ankur Singh, Isha Sharma

**Affiliations:** 1 Department of Psychiatry, University College of Medical Sciences & Guru Teg Bahadur Hospital, Delhi, IND; 2 Department of Ophthalmology, University College of Medical Sciences & Guru Teg Bahadur Hospital, Delhi, IND

**Keywords:** anxiety, coping, depression, rhinorbital mucormycosis, stress, suicidal ideation, visual disability

## Abstract

Aim: The present study addressed overcoming the lacunae in the literature of psychiatric manifestations associated with rhino-orbital mucormycosis. The current study aimed to assess the symptoms of depression, anxiety, stress, coping measures, suicidal intent, and visual disability in patients of rhino-orbital mucormycosis (ROM) during the epidemic of the disease at the nodal tertiary care center in North India.

Methods: Fifty-four inpatients of laboratory-proven rhino-orbital mucor-mycosis (ROM) were included for an observational, cross-sectional study at nodal, designated COVID-19, and mucormycosis treating tertiary care hospital. Patients with Hindi Mental State Examination score <24, prior psychiatric illness, and severely ill requiring ventilator support were excluded. The psychological variables were assessed using Depression, Anxiety, and Stress Scale 21 (DASS 21), Beck’s Suicide intent Scale, Coping Scale Questionnaire, and Visual disability scale (IND-VFQ33). Their socioeconomic status was assessed using the Modified Kuppuswamy Scale.

Results: Ninety percent of patients with ROM had diabetes mellitus. The majority (44%) of patients belonged to lower socioeconomic strata. Higher frequencies of severe depression (28%), extremely severe anxiety (26%), and mild stress (17%) were noted in the study participants. On the Tukey test, depression score was higher in patients of ROM compared to COVID (with ROM) (p-value= 0.016). On Tukey analysis, anxiety score was significantly higher in ROM patients compared to COVID (with ROM) patients (p-value = 0.018). Coping scores were significantly higher in COVID (with ROM) patients compared to ROM patients (p value = 0.035). Mild to moderate visual disability was noted in the study participants.

Conclusion: The current study reflects the association of higher depression and anxiety scores in cases with ROM that indicated higher mental health needs. Early assessment, early detection, and early intervention for psychological help, along with the multidisciplinary team, helped to improve the overall psychological outcome of the affected patients.

## Introduction

Mucormycosis, a ubiquitous fungus belonging to the taxonomic order of Mucorales, is known to cause angio-invasive fungal infection in immunocompromised hosts like patients of uncontrolled diabetes, leukemia, lymphopenia, and organ transplantation [1].

During the pre-COVID era, the annual prevalence of mucormycosis estimated by The Leading International Fungal Education (LIFE) portal was estimated to be around 10,000 cases, but with the inclusion of Indian data, the cases escalated to 910,000 [2]. During the COVID pandemic, the Indian global prevalence of mucormycosis was 70 times higher, with diabetes mellitus emerging as the major risk factor [3].

Due to the sudden surge in cases reaching epidemic levels, the Government of India declared mucormycosis to be a notifiable disease in May 2021. Guru Teg Bahadur hospital (GTBH), the largest tertiary care teaching hospital in East Delhi, was declared a nodal center for the treatment of both COVID-19 cases as well as mucormycosis.

Desai et al. found the following treatment measures, such as control of diabetes mellitus, intravenous infusion of intravenous amphotericin B, and surgical debridement of sinuses, useful for rhino-orbital mucormycosis (ROM) seen in post-COVID-19 [4]. In a recent study carried out on 104 participants in Ireland during the initial phase of the pandemic, rating scales such as Patient Health Questionnaire 9 (PHQ9) and General Anxiety Disorder 7 (GAD 7) were used for the assessment of psychological morbidity. They found that the prevalence of generalized anxiety disorder (GAD) was 20%, and depression was 22.8% in that study. The younger age group, female gender, and low income were associated with the above-mentioned psychiatric morbidity [5].

During the second wave of the pandemic, a web-based online survey was conducted on 500 participants using GAD7, Centre for Epidemiology Scale of Depression. They reported 25.4% of GAD and 18.8% of depressive symptoms, respectively. The study also noted that the younger age group had a higher risk of developing depressive symptoms [6]. In a recent study carried out in 2021, 44.18% presented with post-traumatic stress disorder-like symptoms, 48.8% had significant depression, 65.56% had anxiety, 22.09% had stress symptoms, and 11.27% had disturbed sleep. This study used the Depression, Anxiety, and Stress Scale 21 (DASS 21) for the assessment of psychiatric manifestations. Mental well-being was disturbed in 74.75%, and only 4.15% had high resilience capacity [7].

As per the media reports, a large number of suicides were committed among students probable reason cited was the delay in examination (due to the lockdowns implemented by the authorities to contain the spread of COVID-19), resulting in their career-related uncertainty [8]. The mass movement of farmers, election rallies, religious processions, lack of coordination between center and state level resources, technical expertise, and inequitable distribution of health care facilities in rural and urban areas during the lockdown period probably led to mental health problems in the population [9].

The current study aimed to assess the symptoms of depression, anxiety, and stress along with coping capabilities, suicidal intent, and visual disability in patients of ROM during the epidemic of the disease at the nodal tertiary care center in North India. The early detection and assessment of the psychological manifestations can aid in early intervention.

## Materials and methods

Study design and population

The present study was an observational cross-sectional study jointly conducted by the Department of Psychiatry and Ophthalmology on ROM patients recruited between July 2021 and September 2021 during the second wave of COVID-19 pandemic and the epidemic of mucormycosis reported from India. The study was approved by the institution’s ethics committee and adhered to tenets of the Declaration of Helsinki.

Sample size

To the best of the knowledge of the authors, no study had been conducted before the start of the present study to assess the psychological impact on patients of mucormycosis. Hence, a convenient sample of a minimum of 50 patients with mucormycosis was decided for the present work.

During the study period from July 2021 to September 2021, 450 patients of ROM utilized emergency hospital services. Guru Teg Bahadur Hospital (GTBH), a tertiary care hospital affiliated with a medical school, was turned into a COVID designated facility completely in line with the Government of India guidelines. An additional healthcare facility for less severe cases of COVID-19 was made at Ramlila ground which was also being managed by the staff of GTBH.

**Figure 1:**
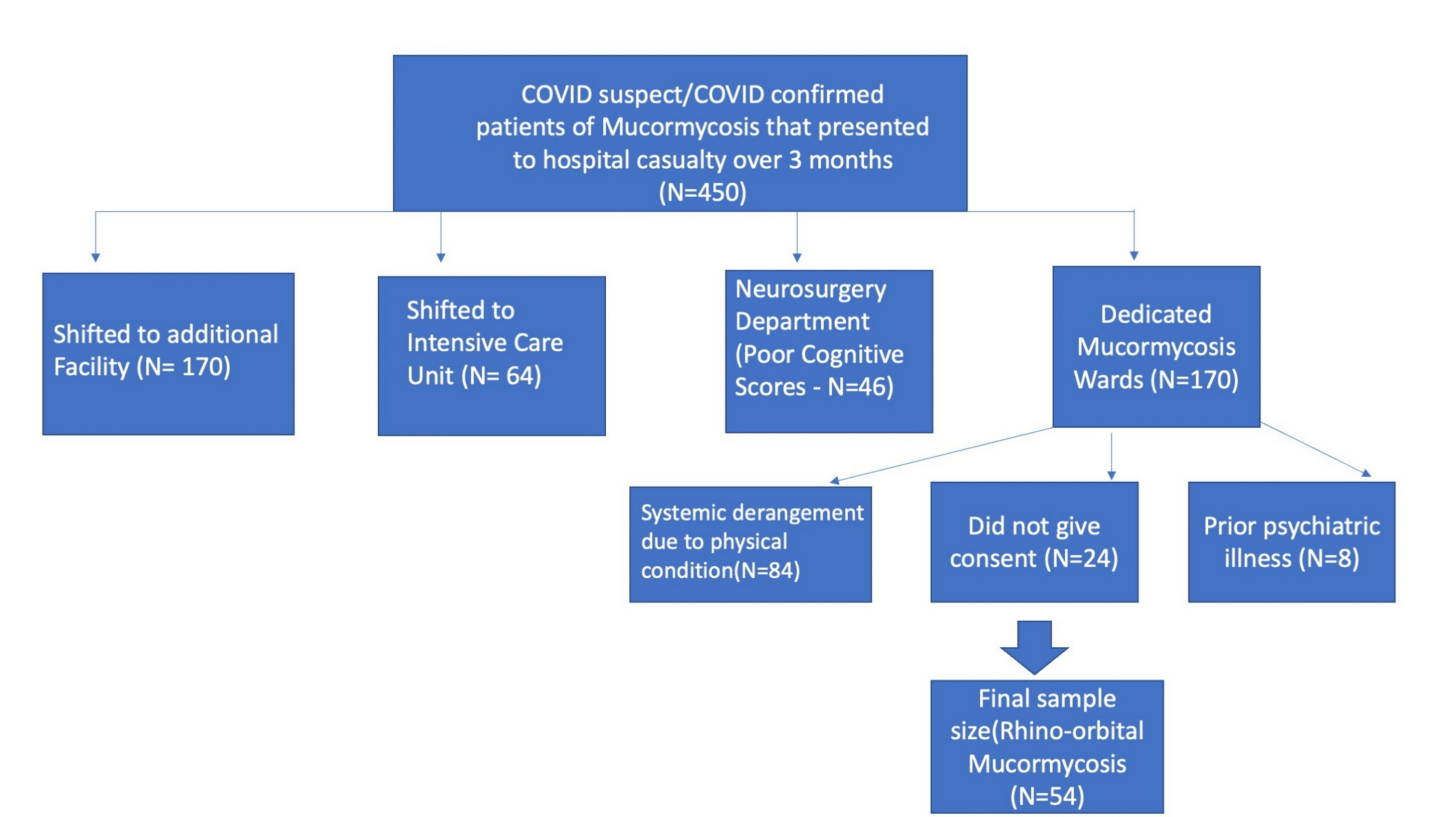
Shows sample size selection of rhino-orbital mucormycosis cases (N=54)

One hundred seventy patients were transferred to an additional healthcare facility. Out of the remaining 280 patients, 64 were shifted to Intensive Care Unit as they required ventilatory support. Eighty-four patients were shifted to the internal medicine department due to severe systemic derangement of electrolytes, blood sugars, or blood pressure, and 46 patients were shifted under the care of the neurosurgery department as they had poor cognitive scores [Hindi Mental Scale Scores, (HMSE) <24]. Eighty-six patients were admitted under the Department of Ophthalmology and Ear, Nose, Throat (ENT), out of which eight were excluded as they had a prior psychiatric illness, and 24 patients did not give consent to participate in the study. Finally, a total of 54 patients were included for analysis in the study (Figure 1).

Data collection and evaluation

A detailed history, including presenting complaints, past history, personal history, family history, socio-demographic history, and detailed physical, mental state, and ophthalmological examination, were carried out for all study participants.

Confirmed COVID-19 positive patients were defined as patients who tested positive for SARS-CoV-2 RNA by reverse transcriptase polymerase chain reaction (RT-PCR). COVID-19 suspects were defined as patients who had clinical symptoms suggestive of COVID-19 but whose RTPCR was negative or who were unable to get tested due to RT-PCR kit constraints.

A patient of mucormycosis was defined as a patient who tested positive by microbiological evidence from scrapings taken by direct nasal endoscopy or tissue biopsy and stained with potassium hydroxide (KOH) stain. All patients were staged according to the staging of Rhino-Orbital-Cerebral mucormycosis given by Honavar et al. [10], who staged the disease according to the site of involvement, signs, symptoms, and diagnostic measures needed for ROM.

Psychiatric questionnaires for assessment of depression, stress, anxiety symptom scale, coping scale, and visual disability scales were applied. The scales were applied within 24-48 hours of admission before the pharmacological intervention with amphotericin B. Cognitive functions were assessed for all participants, and informed consent was obtained.

Hindi version of the Depression, Anxiety, and Stress scale 21 (DASS 21) was used for the assessment of depression, anxiety, and stress symptom scores. Each of the three DASS 21 scales contained seven items divided into subscales with similar content. After the application of the scales, a final score was obtained, which helped to classify patients into normal, mild, moderate, severe, and extremely severe classes of depression, anxiety, and stress [11].

Beck’s suicide intent scale was used to assess suicide intent in patients in a psychometric/measurable way. It had 15 items; and each item was scored from 0 to 2. The final score calculated helped to classify the patients into low, medium, and high risk of suicide [12].

A coping scale questionnaire was used to assess cognitive, emotional, and behavioral methods of dealing with problems in patients with mucormycosis. It comprised 13 items, and each item was scored from 1 to 4. The final score was achieved by adding all the scores. A higher score indicated a higher coping capability [13]. 

Visual Disability scale (IND-VFQ33) had three scales: general functioning (21 items), psychosocial impact (five items), and visual symptoms (seven items). General functioning pertains to a person’s ability to move about, household chores, and activities of daily life on a 5-point scale. Visual symptoms pertained to the effect of quality of life due to visual loss on a 4-point scale. Psychosocial impact pertained to a person’s well-being as well as the quality of social and family interactions on a 4-point scale. A higher score meant poor functionality and, hence, poor vision-related quality of life. This scale was specially designed for the Indian population [14].

HMSE had components of orientation (10 points) which includes temporal orientation (five points) and spatial orientation (five points); Memory (six points) which consisted of immediate recall (three points) and delayed recall (three points); language (eight points) which had naming (two points), verbal repetition (one point), verbal comprehension (three points), writing (one point) and reading a sentence (one point); Attention and calculation (five points) and design copying (one point). The final score was calculated by adding all the scores. A score of 23 or < was taken as a poor cognitive score and, hence, was excluded from the study [15]. All the study participants were classified into three groups. Group 1 had COVID-19 suspect and ROM, group 2 had only ROM, and group 3 had both confirmed COVID-19 and ROM.

Statistical tests

Qualitative data were presented as frequency and percentage. Quantitative data were analyzed using ANOVA and Post hoc Tukey’s test. For statistical significance, a p-value of <0.05 was considered statistically significant. Data analysis was done using IBM Corp. Released 2012. IBM SPSS Statistics for Windows, Version 21.0. Armonk, NY: IBM Corp.

## Results

The study group comprised 54 patients of ROM, with mean age of 53.14 (range 35-70) years. Thirty-four (62.95%) belonged to the male gender, and 20 (37.04%) were females. All study participants (N=54) had a cognitive score of >24 on the administration of the HMSE scale. The mean cognitive score by the HMSE scale for the study participants was 27.35±1.43.

Table 1 illustrates the socio-demographic details of the study patients (N=54). The majority of patients belonged to the upper middle and lower middle classes, according to the modified Kuppuswamy Scale [16]. According to the staging of rhino-orbital-cerebral mucormycosis, 17 (31.48%) patients had sinus with eye involvement (3a). One patient had a history of seizure disorder in the past. No patient in the study had a history of head injury.

**Table 1:** Clinical and sociodemographic profile of the patients of ROM (N=54)

Parameter	Frequency (N=54)	Percentage (%)
Gender		
Male	34	62.95%
Female	20	37.04%
Socioeconomic class		
Upper middle	8	14.81%
Lower middle	22	40.74%
Upper lower	24	44.44%
Occupation of the person		
Professional	5	9.25%
Clerks/Shop owner	1	1.85%
Skilled worker	7	12.96%
Semi-skilled worker	12	22.22%
Unskilled worker	5	9.25%
Unemployed	24	44.44%
Qualification of the person		
Graduate	3	5.5%
Intermediate or diploma	4	7.4%
High school certificate	13	24.07%
Middle school certificate	20	37.04%
Illiterate	14	25.92%
Marital status		
Married	54	100%
Hypertension	20	37.03%
Diabetes	49	90.74%
Beck's suicide intent scale		
Low risk	53	98.14%
Medium	1	1.85%
High risk	0	0%
History of suicide in family	2	3.70%
Staging of disease		
2c	2	3.70%
2d	5	9.20%
3a	17	31.48%
3b	13	24.07%
3c	14	25.92%
4b	3	5.55%
Substance abuse		
Alcohol	3	5.55%
Smoking	6	11.11%

**Figure 2:**
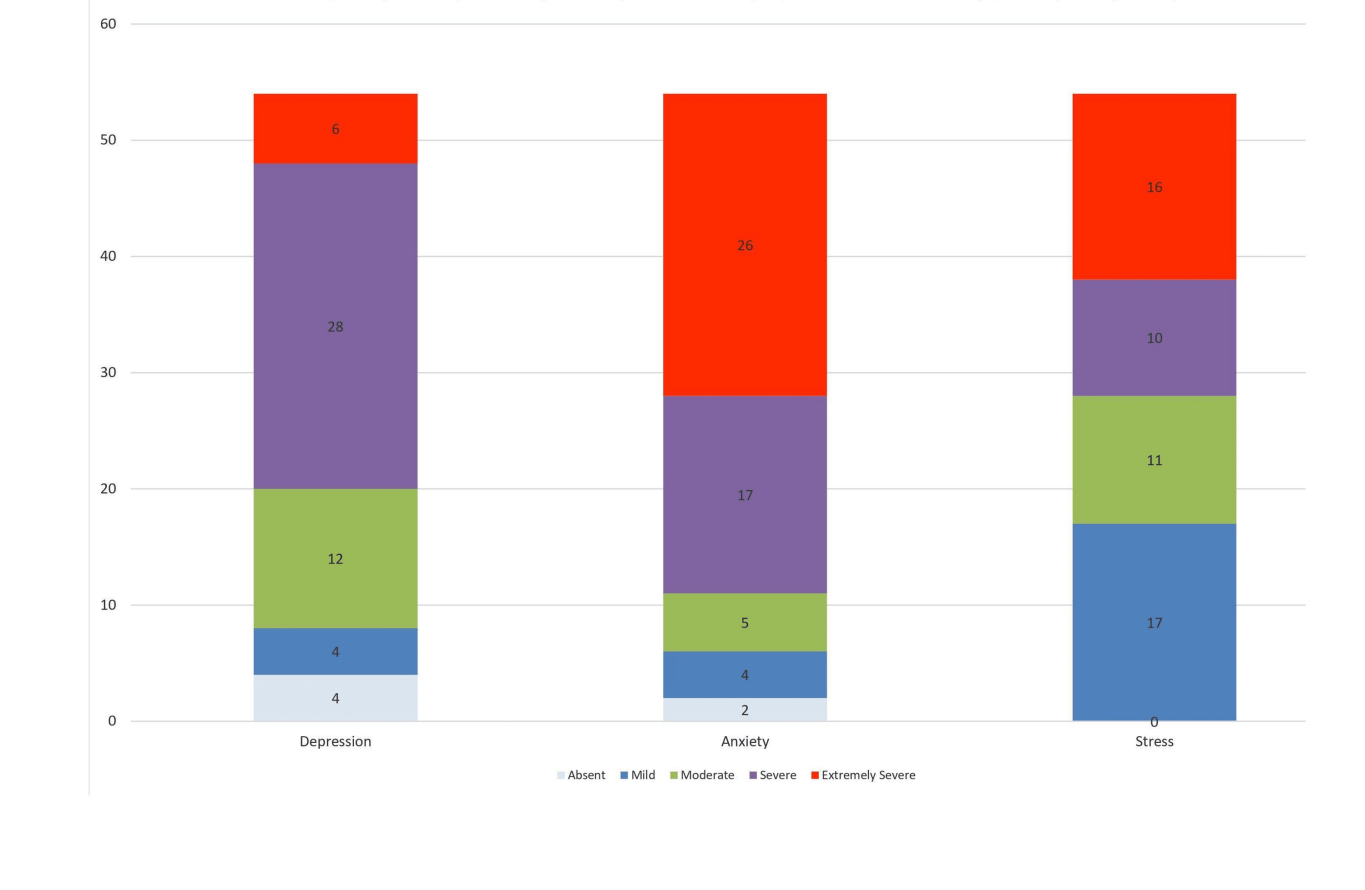
Bar diagram showing the frequency distribution of depression, anxiety, and stress symptom scores in ROM (N=54) The numbers in the figure represent the percentages, ROM: Rhino-orbital mucormycosis

Figure 2 depicts the frequency of depression, anxiety, and stress symptom scores in the study participants (N=54). Higher frequencies of severe depression (28%), extremely severe anxiety (26%), and mild stress (17%) were noted in the study participants.

On comparing depression between three groups; group 1 as COVID suspect & ROM; group 2 as ROM, and group 3 as COVID confirmed & ROM; depression emerged as the statistically significant factor between the three groups (p-value = 0.021) (Table 2).

**Table 2:** Assessment Scales using ANOVA between the three groups, CC+ROM, CS+ROM, only ROM DASS 21: Depression, anxiety, stress score 21; CS: COVID suspect; ROM: Rhino-orbital mucormycosis; CC: COVID Confirmed; VFQ 33: Visual functioning questionnaire 33; GFS: General functioning scale; PIS: Psychosocial impact; VSS: Visual symptom scale; HMSE: Hindi Mental State Examination; * P-value <0.05 taken as significant

Scales	Mean± Standard deviation	ANOVA (p-value)
DASS-21 Anxiety Scores		
CS+ROM	16.40±4.33	
ROM	24.00±8.29	0.012*
CC+ROM	18.21±6.36	
DASS-21 Depression		
CS+ ROM	22.00±6.16	
ROM	23.90±5.46	0.021*
CC+ ROM	19.07±6.02	
DASS-21 Stress		
CS+ ROM	24.80±5.02	
ROM	26.19±8.17	0.940
CC+ ROM	25.93±8.13	
Coping scale		
CS+ ROM	47.80±1.30	
ROM	46.57±1.99	0.040*
CC+ ROM	47.93±1.78	
VFQ-33 (GFS)		
CS+ ROM	26.20±5.49	
ROM	27.29±7.00	0.850
CC+ ROM	27.36±6.93	
VFQ-33 (PIS)		
CS+ ROM	10.00±1.22	
ROM	8.86±1.39	0.400
CC+ ROM	8.93±1.99	
VFQ-33 (VSS)		
CS+ ROM	10.40±1.67	
ROM	10.57±2.29	0.944
CC+ ROM	10.36±2.19	
HMSE score		
CS+ ROM	27.60±1.34	
ROM	27.29±1.15	0.910
CC+ ROM	27.36±1.67	
Age		
CS+ ROM	60.20±7.15	
ROM	52.00±9.24	0.262
CC+ ROM	52.75±11.06	

On Tukey analysis, it was seen that the depression score was higher in group 2 (ROM) compared to group 3 (CC+ROM) (p-value= 0.016). There was no statistically significant difference between other CS+ROM vs. ROM and CS+ROM vs. CC+ROM pairs in relation to depression scores (Table 3).

**Table 3:** Comparison between the groups using post hoc Tukey’s Test , CC+ROM, CS+ROM, only ROM DASS 21: Depression, anxiety, stress score 21; CS: COVID suspect; ROM: Rhino-orbital mucormycosis; CC: COVID Confirmed; VFQ 33: Visual functioning questionnaire 33; GFS: General functioning scale; PI: Psychosocial impact; VSS: Visual symptom scale; HMSE: Hindi mental state examination; *P value < 0.05 taken as significant

Post Hoc Tukey’s Test p-values			
	CS+ROM vs ROM	CC+ROM vs ROM	CS+ROM vs CC+ROM
DASS-21-Anxiety	0.087	0.018*	0.857
DASS-21-Depression	0.789	0.016*	0.558
DASS-21-Stress	0.934	0.993	0.954
Coping scale	0.377	0.035*	0.989
VFQ-33 (GFS)	0.837	0.989	0.873
VFQ-33 (PIS)	0.386	0.989	0.415
VFQ-33 (VSS)	0.987	0.939	0.999
HMSE score	0.902	0.984	0.937
Age	0.242	0.964	0.291

Using ANOVA, anxiety was seen to be statistically significant between the three groups (p-value = 0.012). On further comparison between the groups using Tukey analysis, it was seen that the anxiety score was significantly higher in the MM group compared to the CC+ROM group (p-value = 0.018). There was no statistically significant difference between other CS+ROM vs. ROM and CS+ROM vs. CC+ROM pairs in relation to anxiety parameters (Tables 2, 3).

The coping parameter was seen to be statistically significant between the three groups (p-value = 0.04) using ANOVA. On Tukey analysis, it was seen that the coping scale score was significantly higher in the CC+ROM group compared to the ROM group (p-value = 0.035). There was no statistically significant difference between other CS+ROM vs. ROM and CS+ROM vs. CC+ROM pairs (Tables 2, 3) with respect to coping scale measures.

Between the three groups, the visual disability scale (IND-VFQ33), which included the general functioning scale, psychosocial scale, and visual symptom scale, was not statistically significant. The mean value for the general functioning scale (score range 21-105) was 26.20 for CS+ROM, 27.29 for ROM, and 27.36 for the CC+ROM group indicating that all patients had mild to moderate visual disability while pursuing their daily needs. The mean value for the psychosocial impact scale (score range 5-20) was 10.00 for the CS+ROM group, 8.86 for the ROM group, and 8.93 for the CC+ROM group indicating that mild to moderate disability due to vision affected their psychological and social well-being. The mean value for the visual symptom scale (score range 7-28) was 10.40 for CS+ROM, 10.57 for ROM, and 10.36 for the CC+ROM group indicating the satisfactory quality of life scale measures as measured on the visual disability scale.

## Discussion

The present study was carried out in a tertiary care teaching hospital, which was declared a dedicated COVID care and mucormycosis facility by the Government of India. The majority of the patients recruited in this study belonged to the age group of 35-70 years, with a mean age of 53 years. This is in accordance with the data established by a previous study that compared the epidemiology of mucormycosis in India versus the world. Muthu et al. observed that the mean age of presentation in India was 56 years in comparison with 52 years worldwide [17]. We also observed a male preponderance (62.95%) in the patients who presented to us. This observation goes hand in hand with the existing epidemiological studies on mucormycosis in literature [3,17-20]. 

The majority of patients in this study belonged to the lower socio-economic strata and were unemployed (44.44%), which is in agreement with a study that attributes the preponderance of mucormycosis cases in India to a large population belonging to the lower socioeconomic status [21]. 

Another observation in this study was that a large population of the study were high school graduates. To the best of our knowledge, this factor has not been studied earlier with respect to mucormycosis. All the participants of this study were married, as seen in another study by Ahuja A et al. in 2021 [22]. The risk factors associated with mucormycosis in our patients include diabetes mellitus (90.74%) followed by hypertension (37.03%). The association of these factors has largely been established in the existing literature [23,24].

Steenblock's study [25] reported the interface of COVID-19 with both diabetes and depression, where one condition can predispose to the other condition. One of the risk factors associated with COVID-19 survivors is the development of new-onset DM due to deranged blood sugar levels and viral infiltration of islets of the pancreas [25]. Steenblock's study [25] reported that both COVID-19 and diabetes mellitus are associated with insulin resistance and the role of inflammation. A recent study reported higher levels of inflammatory markers such as C reactive protein and Neutrophil-Lymphocyte ratio in the depressive-suicidal patient group [26]. All three conditions, namely COVID-19, diabetes, and depression, as per the previous studies mentioned above, are associated with inflammation, although the causative role is yet not established.

The majority of the participants were classified as Stage 3 and above. This is also in accordance with a multi-centric Indian study published in 2021 [18]. The sound methodology and standard rating scales, larger sample size, availability of amphotericin B injections during the pandemic, and dedicated multidisciplinary treating team were the strengths of this study.

Previous evidence, literature, and media highlighted the need for assessment of suicidal risk, as the pandemic overall had set in a gloomy picture [5-9]. The finding of 98% cases of low suicide risk could be explained by the regular counseling services which were provided for all the patients after initial evaluation. One patient reported high suicidal risk (who had a history of suicide in the family) and was clinically diagnosed as suffering from grief reaction with a depressive episode (ICD F43). Selective serotonin reuptake inhibitor (SSRI), Fluoxetine 10 mg per day, was administered along with regular counseling sessions. Another study by Ahuja et al. [22] did not report any significant association of COVID-associated mucormycosis with suicidal ideation.

The study sample comprising ROM had stress symptoms in 100% of patients and depressive and anxiety symptoms in 92% and 96%, respectively. The depression (p-value = 0.021) and anxiety (0.012) symptoms were significantly associated with COVID suspect (with ROM), COVID (with ROM), and only ROM. Previous studies also report a higher association between depression and anxiety symptoms during the COVID-19 pandemic [6-7]. A previous study by Ahuja et al. [22] reported that 76.4% of patients were experiencing anxiety or sadness before surgical intervention.

Our study was unique in highlighting the presence of Rhino-Orbital Mucormycosis (N=54) with higher mean scores of both depression and anxiety symptoms which was like the previous study by Nair et al. [27] that consisted of 64% of a sample of the cutaneous type of mucormycosis. The post hoc Tukey test also confirmed the higher scores of depression (p-value = 0.016) and anxiety (p value=0.018) in only ROM. The confirmed laboratory presence of ROM alerted the clinicians in collaborating with the departments of endocrinology as well as psychiatry in an inpatient setting at an early stage for providing comprehensive management.

Poorer coping methods were associated with ROM (p value= 0.035) in comparison with COVID cases in the study sample. This could be explained by the visual disability symptoms observed more in ROM cases that lead to impairment of day-to-day activities. One of the possible mechanisms for the higher association of depression and anxiety symptoms in ROM cases could be attributed to underlying physical ailments such as blindness in one eye, diabetes mellitus, hypertension, and higher stage of ROM 3 or above in the present study.

Psychiatry consultations were made a norm at the COVID and mucormycosis designated study center for all ROM admitted patients, in liaison with the other departments managing the disease as a part of the multi-disciplinary team. Appropriate interventions involving both pharmacological measures and counseling/psycho-educative support sessions were executed for all screened positive ROM cases (after the application of scales to assess their psychological state) by a trained senior psychiatrist who had expertise in dealing with COVID-19/mucormycosis cases.

Follow-up counseling sessions were exclusively conducted in association with the department of ophthalmology to deal with patients with mild to moderate visual disability. This is the first study in the literature to assess visual disability due to mucormycosis in terms of how it affects their general functioning, social life, and its effect on quality of life. Apart from the study cases, psychological support sessions were also conducted for their primary caregivers for educating them on how to handle the patients and help them deal with the post-discharge complications/interventions of both COVID and ROM. The limitation of this study is that the results cannot be extrapolated to the entire Asian region, as the data is obtained from a nodal tertiary center in North India dealing with the cases of COVID and ROM. Further research work requires pooled data from different regional centers involving different geographical locations.

## Conclusions

Nevertheless, the current study reflects the association of higher depression and anxiety scores in cases with ROM, which emphasizes the need for early intervention to improve the mental health as well as the overall well-being of the affected patients. Early assessments, regular counselling sessions may have contributed to the low suicide risk in ROM cases. The study enabled early detection of cases with higher depression, anxiety, stress symptoms, poor coping methods, and visually disabling symptoms resulting in poor psychosocial and general functioning. Early intervention by trained mental health professionals in liaison with a dedicated multidisciplinary team of medical experts improved the overall outcome of these ROM cases. 
