# Correction: The Psychological Impact of Rhino-Orbital Mucormycosis During the Second Wave of COVID-19 Pandemic From South East Asian Country

**DOI:** 10.7759/cureus.c368

**Published:** 2025-10-24

**Authors:** Shruti Srivastava, Nitika Beri, Gopal K Das, Pramod K Sahu, Ankur Singh, Isha Sharma

**Affiliations:** 1 Department of Psychiatry, University College of Medical Sciences & Guru Teg Bahadur Hospital, Delhi, IND; 2 Department of Ophthalmology, University College of Medical Sciences & Guru Teg Bahadur Hospital, Delhi, IND

This article has been corrected to clarify that the authors utilized the Hindi Mental State Examination (HMSE), not the Hindi Mini-Mental State Examination. All references to the Mini-Mental State Examination (MMSE) have been corrected.

